# The effectiveness, safety, and economic evaluation of Korean medicine for unexplained infertile women: A multi-center, prospective, observational study protocol

**DOI:** 10.1097/MD.0000000000009360

**Published:** 2017-12-22

**Authors:** Su-Hyun Kim, Junyoung Jo, Dong-Il Kim

**Affiliations:** a Graduate School of Korean Medicine, Dongguk University, Gyeongju; b Conmaul Hospital of Korean Medicine, Seoul; c Department of Korean Obstetrics and Gynecology, Dongguk University Ilsan Hospital of Korean Medicine, Goyang, Republic of Korea.

**Keywords:** female infertility, live birth, pregnancy rate, traditional Korean medicine

## Abstract

Infertility is a condition in which a woman has not been pregnant despite having had normal intercourse for 1 year. The number of unexplained infertile females is increasing because of late marriage customs, as well as environmental and lifestyle habits. In Korea, infertile females have been treated with Korean medicine (KM). However, these effects have not been objectively confirmed through clinical trials. Therefore, this study was conducted to demonstrate the effectiveness of herbal medicine treatment in infertile patients and to demonstrate the economic feasibility through economical evaluation with assisted reproductive technology.

This study is designed as a multicenter, single-arm clinical trial. All participants included will be from 3 Korean Medicine hospitals in Korea and will voluntarily sign an informed consent agreement. All recruited patients will conduct related surveys and tests, and be provided with treatment according to their menstrual cycle. Patients will take herbal medicines for 4 menstruation cycles and receive acupuncture and moxibustion treatment at 3 times (menstrual cycle day 3, 8, 14) during 4 menstruation cycles. They will also undergo an approximately 4 menstrual cycle treatment period, and 3 menstrual cycle observation period. If pregnant during the study, participants will take the herbal medicine for implantation for about 15 days. In this study, the primary outcome will be the clinical pregnancy rate, whereas the secondary outcome will include the implantation rate, ongoing pregnancy rate, and live birth rate.

Ultimately, this study will provide clinical data regarding the effectiveness and safety of KM treatment for females with unexplained infertility and important evidence for establishing standard KM treatments for unexplained infertility. Moreover, we will identify the most cost-effective way to treat unexplained infertility.

## 1 Background

Infertility is a condition in which successful pregnancy has not occurred, despite normal intercourse over 12 months. Approximately, 9 to 18% of normal couples are infertile.^[1]^ In recent years, the number of elderly pregnancies has been increasing because of changing social trends. Moreover, infertile females are increasing due to stress, obesity, lack of exercise, and environmental pollution.^[2]^ The causes of female infertility are classified as ovulation factors, tubal and peritoneal factors, cervical factors, uterine factors, immunological factors, infection factors, and unexplained. Among these, unexplained infertility is estimated to occur in about 15% of patients.^[3,4]^

Since 2006, Korea has begun to support project medical expenses for infertility. Over the last 10 years, the supporting project for assisted reproductive technology (ART) has grown quantitatively, bringing about 32,000 in vitro fertilizations (IVF) and 30,000 intrauterine insemination (IUI) treatments annually. The total cost of the project is 85.7 billion won; however, the clinical pregnancy rate (CPR) is about 33.5% for IVF and 10% for IUI, which is not very different from 10 years ago.^[5,6]^

Korean medicine (KM) has long utilized acupuncture and herbal medicine treatment for the treatment of infertility.^[7]^ Specifically, KM is widely used as a primary or adjuvant therapy of infertility in Korea and to improve the success rate of ART.^[8–10]^ Many investigations of KM treatment for infertility have been conducted in Korea; however, most investigations of KM treatment were experimental studies, case reports, retrospective chart reviews, and questionnaire studies.^[11,12]^ Several projects recently conducted by local governments and the association of Korean medicine revealed that the results of KM to treat infertility were comparable with IUI.^[7,8,13,14]^ However, many of these studies did not employ standardized KM treatments for unexplained infertility. Moreover, very few studies have been conducted to collect evidence of KM treatment effects on unexplained infertility.^[15]^

Therefore, we have developed a protocol for presenting standard KM treatment of infertility, as well as confirming the effectiveness and safety of KM treatment of unexplained infertility and providing an economic evaluation when compared with ART.

## 2 Methods/design

### 2.1 Study aims

This clinical trial was conducted to confirm the clinical effectiveness and safety of KM treatment of unexplained infertility and to evaluate the cost-effectiveness relative to ART.

### 2.2 Design/setting

This study was designed as a multicenter, single-arm prospective clinical trial. All participants included will be from 3 hospitals: Dongguk University Ilsan Korean Medicine Hospital, Kangdong Kyunghee Korean Medicine Hospital, and Wonkwang University Kwangju Korean Medicine Hospital. Participants will be recruited through advertisements on bulletin boards at each medical center. The total target sample size is 100 subjects. All participants will voluntarily sign an informed consent agreement. Moreover, all participants will be enrolled after screening via the inclusion/exclusion criteria. All recruited patients will perform related surveys and tests, and be provided with treatments (herbal medicine, acupuncture, moxibustion). They will undergo an approximately 4 menstrual cycle treatment period and a 3 menstrual cycle observation period. If pregnancy occurs during the study, we will offer the herbal medicine to participants for about 15 days. The costs spent (transportation, time, care) in the previous treatment of the participants of this clinical trial will be investigated and compared with the conversion cost of this trial.

### 2.3 Recruitment

Participants will be recruited through advertisements on bulletin boards at each medical center. The target sample size is 100 subjects. Screening will be performed before the trial to determine if patients fulfill the selection criteria. Final enrollment will be determined by survey of menstrual history, medical history, and questionnaire for infertility treatment.

### 2.4 Eligibility criteria

#### 2.4.1 Inclusion criteria

(1) Female over 20 and under 44 years of age;
(2) Unexplained infertile female with a medical certification from a doctor of obstetrics-gynecology;
(3) Has sexual intercourse more than twice a week.

#### 2.4.2 Exclusion criteria

(1) Unable to take herbal medicine, acupuncture, and moxibustion treatment during 4 menstruate cycle treatment periods;
(2) Unable to participate in this clinical trial for 7 months;
(3) Takes pregnancy-related medicine (e.g., hormones, other herbal medication, etc);
(4) Planning to undergo artificial insemination or IVF;
(5) A has irregularly menstruating occurring at intervals of less 21 days or more than 40 days
(6) Male factor infertility;
(7) Has ovulation factor infertility (polycystic ovary syndrome, thyroid disease, hyperprolactinoma, etc);
(8) Has tubal or peritoneal or uterus factor infertility (endometriosis, pelvic inflammatory disease, uterine myoma, adenomyosis, etc);
(9) Is diagnosed with dampness heat, kidney yin deficiency;
(10) Has taken IVF than 4 times;
(11) Has severe neuropsychological disease history;
(12) Has a severe disease related to infertility (e.g., hypertension, diabetes, chronic/acute hepatitis, hyperlipidemia, cardiovascular disease, cancer, chronic/acute inflammatory disease, etc);
(13) Has cancer history within 5 years;
(14) Has a skin disease over an acupuncture/moxibustion point;
(15) Has an allergy or hypersensitivity to treatment (herbal medicine, acupuncture, moxibustion);
(16) Is planning to participate in another clinical trial within 3 months;
(17) Does not have the ability to fill out forms.

### 2.5 Interventions

#### 2.5.1 Herbal medicine

All participants will take the herbal medicine according to their menstrual cycle. The detailed herbs in the prescriptions are listed in Table 1. Onkyeong-tang is a popular prescription in Donguebogam that regulates the menstrual cycle and infertility.^[16]^ The prescription for ovulation and implantation has been made and used by authors from Dongguk University Ilsan Korean Medicine Hospital.^[17]^ This prescription was made by Dong-il Kim on the basis of Yuklin-ju, Ontoyuklin-ju and Sutae-hwan. The 2 prescriptions have been used to investigate infertility over 3 years and 200 cases, and have therefore received consent for use as medication for clinical trials from the Society of Korean Medicine Obstetrics and Gynecology. Onkyeong-tang will be decocted 30 packs (120 cc/pack) and participants will be instructed to take the medicine orally twice daily between menstrual cycle day (MCD) 3 and 12. The prescription for ovulation and implantation will be decocted 30 packs (120 cc/pack). Participants will be instructed to take medicine orally twice daily between MCD 13 and 28 every menstrual cycle. All herbal medicines will be manufactured by Jaseng-medicinal decoctions outside of the hospital, where there are semi-good manufacture practice (GMP) facilities and GMP herbs are used.

**Table 1:**
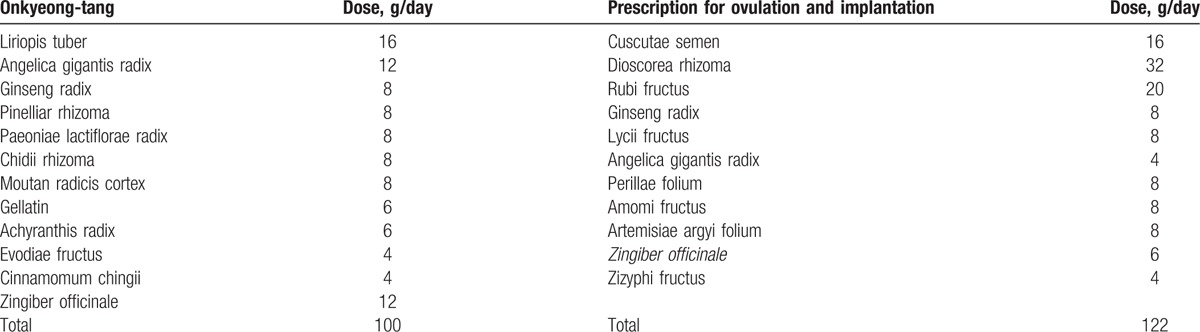
Contents of Onkyeong-tang and prescription for ovulation and implantation.

#### 2.5.2 Acupuncture and moxibustion

All patients will receive acupuncture treatment on 14 acupoints, GV20, CV4, bilateral Ex-CA7, ST29, SP6, SP9, ST36, and PC6 using acupuncture needles 0.3 mm in diameter and 30 mm in length (Dong Bang, Gyeonggi-do, Korea). At GV20, practitioners will insert needles 5 mm into the scalp fascia. At other acupoints, needle insertion will be to a depth of 15 to 20 mm, depending on the region of the body into which the needles were inserted. These procedures will elicit de-qi sensation. We will use electro-acupuncture for maintain de-qi and infrared therapy to heat the lower abdomen. The electro stimulation will be administered for 20 minutes at low-continuous 2 Hz. Following elicitation of the de-qi sensation at CV4, we will apply moxibustion for 20 minutes. Acupuncture and moxibustion treatments will be performed at MCD 3, 8, and 14 during 4 menstruation cycles. If the patient becomes pregnant during treatment, these treatments will be stopped.

### 2.6 Outcome measures

#### 2.6.1 Primary outcome

The primary outcome of this study will be CPR. Clinical pregnancy was considered to be at least 1 gestational sac with fetal heart activity confirmed by ultrasound scan 5 to 6 weeks after pregnancy.^[18]^

#### 2.6.2 Secondary outcome

The secondary outcome of this study will be implantation rate (IR), ongoing pregnancy rate (OPR), and live birth rate. IR is confirmed by at least 1 gestational sac after human chorionic gonadotropin positive.^[19]^ Ongoing pregnancies are defined as the presence of fetal cardiac activity beyond 12 weeks of gestation.^[20]^ OPR is defined as the number of ongoing pregnancies divided by the number of clinical pregnancies. Live birth is defined as the delivery of 1 or more living infant (have heart beats or have beats of umbilical cord or have definite movement of voluntary muscles).

#### 2.6.3 Sample size

We did not follow the general method to calculate sample size. The sample size of this study was determined by Ministry of Health and Welfare and Korean Health Industry Development Institute in accordance with study budget, period, and purpose. A total of 100 participants will be recruited, of which 5 will be 20 to 29 years old, 40 will be 30 to 35 years old, 40 will be 35 to 39 years old, and 15 will be 40 to 44 years old. The detail sample size is shown in Table 2, the number of participants by age is based on report of age of the National Supporting Program for Infertile Couples Analysis and Evaluation of supporting project for infertile couples in 2012.^[21]^ Age distribution of participants supported by IVF is shown in Table 3.

**Table 2:**
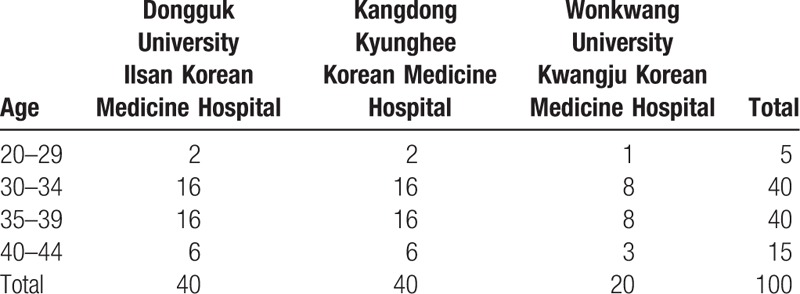
Sample size according to age group.

**Table 3:**
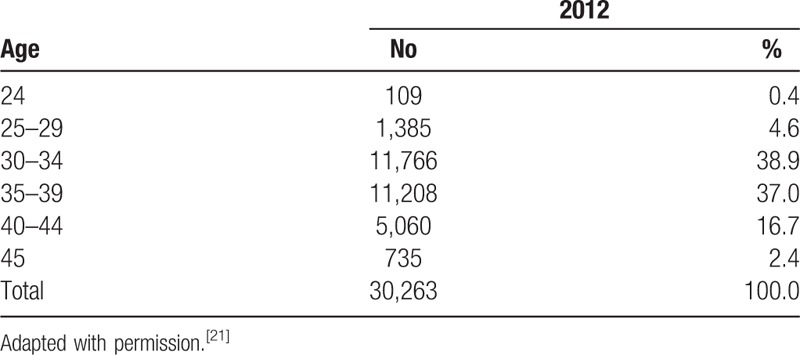
Age distribution of participants supported by IVF.

### 2.7 Study visit and data collection

#### 2.7.1 In case of pregnancy failure

The clinical study flow chart in case of pregnancy failure is shown in Table 4.

**Table 4:**
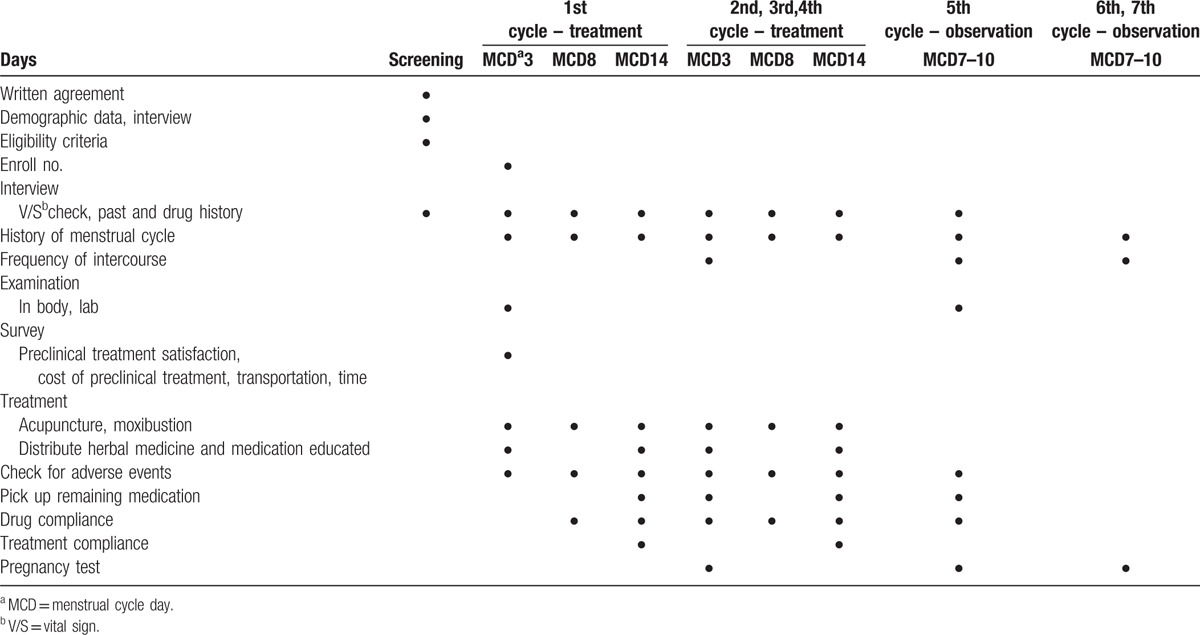
Study flow chart in case of pregnancy failure.

#### 2.7.2 In case of pregnancy success

The clinical study flow chart in case of pregnancy success is shown in Table 5.

**Table 5:**
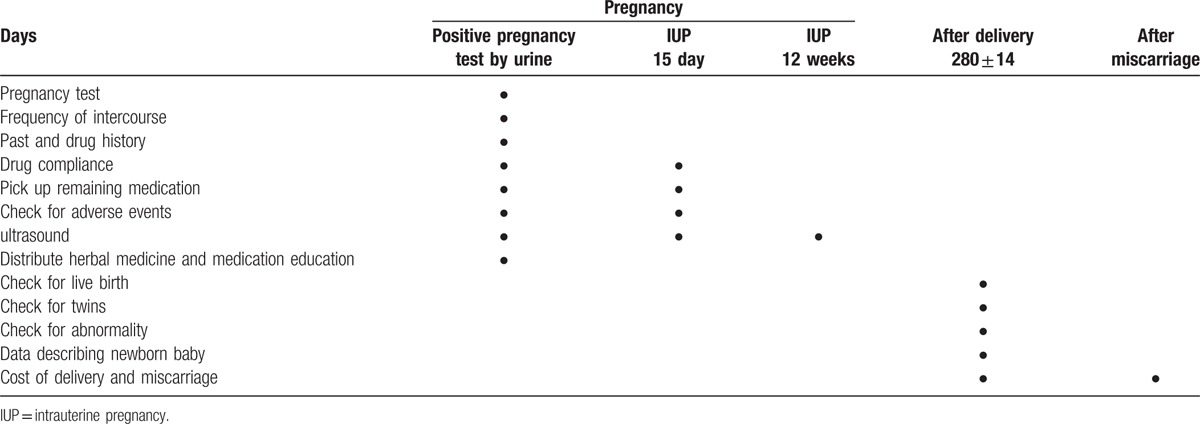
Study flow chart in case of pregnancy success.

### 2.8 Statistical analysis

The analysis set will comprise a full analysis set and a per-protocol set. Descriptive analyses including the mean, standard deviation, and frequency will be undertaken describing the baseline characteristics of participants. Continuous variables of primary and secondary outcomes will be presented in descriptive statistics and categorical data will be presented in frequency and percentage. When comparing and analyzing according to the level of specific variables such as demographic variables, continuous data will be tested using a Student's *t*-test or Wilcoxon's rank sum test. Categorical data will be analyzed using a chi-squared test or Fisher's exact test. For before and after comparisons, a paired *t*-test or Wilcoxon's signed rank test will be used. Repeated measures ANOVA will also be performed, whereas McNemar's test will be performed on categorical data. All statistical analyses will be conducted at a significance level of 0.05 and tested on both sides. Statistical analysis will be performed using the SAS program (version 9.4; SAS Institute Inc., Cary, NC).

#### 2.8.1 Missing data

Upon analysis of the validation variables, if the data are missing at some point or the subject is missing, the last observation carried forward (LOCF) analysis will be performed as if it were obtained at that time. The LOCF analysis applies to all validation analyses.

### 2.9 Additional analysis

#### 2.9.1 Safety evaluation

At each visit, any subjective and objective discomforts will be collected through interviews, after which these will be recorded on patients case report form (CRF). The clinical laboratory tests will be performed before and after the treatment period to check for hematologic problems.

#### 2.9.2 Economic evaluation

Economic evaluation will be conducted from a social perspective and all costs and results will be considered. The cost data per patient in this trial will be requested to submit medical institutions or card receipts for expenses incurred in the preclinical treatment. If patients cannot submit a receipt, we will conduct a survey of preclinical treatment cost. The cost of this trial will be calculated by converting this trial treatment schedule into a general treatment. Transportation costs and time costs will be used to existing values that patients had spent, and surveys will be conducted during the clinical trial period.

### 2.10 Quality assurance

We will set qualification standards to make sure that patients are treated in according with this trial. Therefore, we will inform all practitioners of the details of this trial. Treatment will be performed by doctors of Korean medicine who have extensive clinical experience with acupuncture and moxibustion. Specifically, they will be graduates of a 6-year full-time course in Korean Medicine, taught as a college program, certified by the Korean Ministry of Health and Welfare as a Korean Medicine Doctor, and have more than 1 year of postgraduate clinical training in a Korean Medicine hospital. Compliance of herbal medicine, acupuncture, and moxibustion should be in the range of 80% to 120% per visit. In this trial, only subjects with compliance of 70% or more will continue the study.

### 2.11 Adverse events

Any physical or clinical changes will be reported by practitioners, coordinators, and patients in the CRF at each visit and/or by telephone. All related symptoms, date of onset, and duration will be recorded in the CRF. If an adverse event is severe and associated with the trial, we will provide appropriate medical care, and the participant may be withdrawn from this study in such cases. In the case of herbal medicines, slight digestive disorders may occur in some patients. In relation to herbal medicines, some digestive disorders may appear in some patients.^[22]^ In acupuncture treatment, some patients may have mild bleeding and ecchymosis.^[23,24]^ Moxibustion treatment may result in erythema, mild burns, and allergic reaction in some patients.^[24,25]^ However, there are no expected severe adverse events, which are defined as death, life-threatening adverse experiences, hospitalization or prolongation of existing hospitalization, persistent or significant disability/incapacity, or the potential for a congenital anomaly/birth defect resulting from pregnancy.

### 2.12 Ethics

This study protocol was externally peer reviewed by the Korean Health Industry Development Institute. Additionally, the trial was authorized by the Institutional Review Board of Dongguk University Ilsan Korean Medicine Hospital (approval number: 2016-01), Kangdong Kyunghee Korean Medicine Hospital (approval number: KHNMCOH2016-04-004-002), and Wonkwang University Kwangju Korean Medicine Hospital (approval number: 2016/1). All recruited patients will provide informed consent before enrollment. This trial is registered with the Korean Clinical Trial Registry (CRIS), Republic of Korea: KCT0002235.

## 3 Discussion

Recently in Korea, infertile patients have been highly dependent on ART. Since the government supports the cost of ART based on income from 2006,^[26]^ the number of patients who select ART are increasing annually.^[27]^ The number of infertile patients who are supported by the cost is increasing, as is the financial burden borne by the government. However, the pregnancy rate and success rate of ART are at a standstill. Furthermore, there are side effects associated with ART. During ART, ovarian hyper-stimulation is often performed using an ovulation inducer, which causes ovarian hyperstimulation syndrome (OHSS).^[28,29]^ As the number of infertile patients using ART increases, the incidence of OHSS is also increasing.^[30]^ Previous studies have also shown that maternal complications and adverse pregnancy outcomes such as preterm labor, premature birth, and low birth weight are increasing because of twin pregnancies after ART.^[31]^

Since ancient times, Korea has been influenced by Confucianism; therefore, it has been important to focus on the blood-centered social structure. Pregnancy and delivery have been the greatest factors in supporting the blood-centered society.^[32]^ Accordingly, in Korea, the treatment of infertility using herbal medicines, moxibustion, and acupuncture has been developed. Recently, KM therapies have been confirmed to be effective at improving the general function, endocrine, and reproductive function of women, and they are currently being used as supplementary therapy in addition to ART.^[14]^

However, no clinical trials have confirmed the effectiveness and safety of standard KM therapies for infertility. Because of this, KM has not been recognized as a standard treatment for infertility, and it has been excluded from national projects.

In this study, we will set up standard prescriptions according to the menstrual cycle on the basis of various studies and the prescriptions applied for infertility in Korea's representative large Korean medicine clinics. The standardized comprehensive treatment that can be applied to infertile women will be set up by combining herbal medicine, acupuncture and moxibustion. Overall, the ultimate purpose of this trial is to evaluate the effectiveness and safety of KM treatment for unexplained infertility and to evaluate the cost-effectiveness relative to ART. A total of 100 patients will be classified by age group, after which they will receive herbal medicine treatment according to patient's menstrual cycle and the CPR, IR, OPR, and live birth rate will be evaluated. To confirm the safety of KM treatment, we will check for adverse events every visit. Moreover, to evaluate cost-effectiveness, we will compare the economical effectiveness with that of ART.^[33]^

It should be noted that this trial has limitations. Specifically, it is based on a single treatment group without a blind, randomized control group. Because people usually do not want to wait to conceive, it is not easy to set up a control group. Therefore, literature pertaining to ART will be collected and used to replace a control group to compare the effectiveness and cost-effectiveness. This study is funded by the Ministry of Health and Welfare and the Korea Health Industry Development Institute and the results will be grounds for national policy projects. Moreover, to compare the effectiveness and economics of ART, the recruitment was based on the percentage of assisted reproductive costs supported in the country in 2010.

Despite these limitations, this clinical trial is the first to obtain an objective basis for the effectiveness and safety of KM treatment. The cost comparison with ART is expected to confirm the economic feasibility of Korean medicine treatment, which will allow us to offer a new direction in the treatment of infertility treatment, which is biased toward ART in Korea. We also expect it to be used globally as an alternative to solve the unexplained infertility.

### 3.1 Trial status

Patient recruitment is ongoing. Patient enrollment and trial completion is expected by May of 2018.
