# The effectiveness, safety, and economic evaluation of Korean medicine for unexplained infertile women: A multi-center, prospective, observational study protocol: Erratum

**DOI:** 10.1097/MD.0000000000009682

**Published:** 2018-01-19

In the article, “The effectiveness, safety, and economic evaluation of Korean medicine for unexplained infertile women: A multi-center, prospective, observational study protocol”,^[1]^ which appeared in Volume 96, Issue 51 of *Medicine*, affiliations a and b are missing some information. They should appear as:

^a^ Department of Korean Obstetrics and Gynecology, Graduate School of Korean Medicine, Dongguk University, Seoul, Republic of Korea

^b^ Department of Korean Obstetrics and Gynecology, Conmaul Hospital of Korean Medicine, Seoul, Republic of Korea.
